# Patient Factors Associated with Presacral Space Depth: A Pelvic MRI Study

**DOI:** 10.1007/s00192-026-06645-0

**Published:** 2026-04-16

**Authors:** Krystine Gulbrand, Amanda Chapa, Mariana Masteling, Jordan Swensson, Anne Sammarco, Ramzy Burns, Douglass Hale, John D. E. Lancey, Jennifer Hamner

**Affiliations:** 1 Division of Urogynecology, Indiana University School of Medicine, Indianapolis, IN USA; 2 Department of Mechanical Engineering, University of Michigan, Ann Arbor, MI USA; 3 Department of Radiology, Indiana University School of Medicine, Indianapolis, IN USA; 4 Department of Obstetrics and Gynecology, University Hospitals, Cleveland, OH USA; 5 Department of Obstetrics and Gynecology, University of Michigan, Ann Arbor, MI USA; 6 Present Address: Department of Urology, Indiana University School of Medicine, Indianapolis, IN USA

**Keywords:** Hysterectomy, MRI, Pelvic anatomy, Pelvic organ prolapse, Presacral space, Sacrocolpopexy

## Abstract

**Introduction and Hypothesis:**

Sacrocolpopexy is the most durable surgical repair for advanced pelvic organ prolapse, but dissection of the presacral space can be technically challenging. Variation in presacral space depth may affect surgical exposure and operative risk. Limited data exist regarding patient-specific factors associated with variation in presacral anatomy and presacral space depth. We hypothesized that body mass index (BMI) would not meaningfully predict variation in presacral space depth.

**Methods:**

This retrospective cohort study evaluated presacral anatomic measurements using pelvic magnetic resonance imaging (MRI) in women. Presacral anatomic measurements, including presacral depth, anterior longitudinal ligament thickness, and sacral vertebral dimensions, were obtained between L5 and S3 using a standardized midsagittal MRI technique. Patient demographics, clinical characteristics, and medical history were collected. Descriptive statistics, correlation analyses, and effect sizes were calculated.

**Results:**

A total of 209 pelvic MRIs were reviewed, of which 167 had complete data. BMI demonstrated only weak correlations with presacral space depth. A history of hysterectomy was associated with increased presacral depth at S1 compared with women with an intact uterus (24% increase; *p* = 0.01). Postmenopausal status and exogenous estrogen use were associated with increased L5–S1 disc space height (*p* = 0.03 and *p* = 0.02, respectively). Presacral space depth was significantly shallower at the sacral promontory than at more caudal levels, while anterior longitudinal ligament thickness was greatest at the promontory. Presacral depth was also moderately correlated with sacral vertebral body height.

**Conclusions:**

Presacral space depth was not associated with BMI but was modestly increased in women with a history of hysterectomy. These findings suggest that prior pelvic surgery, rather than BMI, may influence presacral anatomy and could be relevant for surgical planning.

## Introduction

Pelvic organ prolapse (POP) is a common condition, with an estimated lifetime risk of primary prolapse surgery of 12.6% by age 80, increasing steadily with advancing age [1]. Abdominal sacrocolpopexy remains the most durable surgical operation for advanced POP and is widely regarded as the gold standard for comparison [2, 3]. A key component of the procedure is dissection of the presacral space to the anterior longitudinal ligament (ALL) for mesh fixation.

Pelvic organ prolapse (POP) is a common condition, with an estimated lifetime risk of primary prolapse surgery of 12.6% by age 80, increasing steadily with advancing age [1]. Abdominal sacrocolpopexy remains the most durable surgical operation for advanced POP and is widely regarded as the gold standard for comparison [2, 3]. A key component of the procedure is dissection of the presacral space to the anterior longitudinal ligament (ALL) for mesh fixation.

The presacral space is a retroperitoneal region bordered by the peritoneum superiorly, the pelvic floor inferiorly, and the internal iliac vessels laterally [4]. It contains loose areolar tissue, vascular structures, autonomic nerves, and adipose tissue, which likely represents perivascular fat surrounding presacral vessels and is more similar to subcutaneous adipose tissue than mesenteric or visceral fat [5]. Increased presacral depth, measured as the distance between the posterior peritoneum and the ALL, can make dissection more challenging and increase risk associated with sacrocolpopexy. Although rare, severe presacral hemorrhage has been reported, and venous bleeding is challenging to control in this space due to high hydrostatic pressure and the absence of venous valves [6]. Additionally, cadaveric studies have shown that the hypogastric nerve is at risk during presacral dissection [7]. Ripperda et al. described the intricate anatomy of the inferior hypogastric plexus, offering insight into potential reasons for postoperative visceral and sexual dysfunction [5]. The presacral space is accessed not only in sacrocolpopexy, but also in colorectal, orthopedic, and oncologic procedures.

Limited data exist regarding patient factors that may influence presacral anatomy. Prior work has shown that men have a greater presacral depth than women, but additional patient characteristics that may contribute to this variation have not been well studied [8]. Camhi et al. demonstrated that BMI did not correlate with visceral adipose tissue deposition, particularly in older adults [9]. Because BMI reflects overall body mass rather than the distribution of adipose depots, including subcutaneous, visceral, and perivascular fat, it may not accurately predict localized anatomic variation within retroperitoneal spaces such as the presacral region. Despite this, BMI is frequently assumed to influence the technical difficulty of retroperitoneal dissection during pelvic surgery. Given that variation in presacral space depth, vertebral morphology, and the position of vascular and neural structures may affect surgical exposure and operative risk, identifying patient factors associated with presacral anatomy may improve preoperative planning.

The primary objective of this study was to evaluate patient characteristics, including BMI, age, race, POP status, and relevant medical and surgical history that may correlate with presacral anatomic measurements on MRI, including presacral space depth, ALL thickness, and sacral vertebral dimensions. A better understanding of these relationships may improve knowledge of presacral anatomy, support surgical planning, and ultimately enhance outcomes for procedures involving the presacral space.

## Materials and Methods

In this study, we utilized the program 3D Slicer (https://www.slicer.org/) to conduct measurements in the midsagittal plane of MRI images sourced from the University of Michigan Pelvic Floor Research Group MRI database (OPAL2 Dataset IRB# HUM00043445, IRB # 14,319). All measurements were obtained in the midsagittal plane using the slice demonstrating the clearest visualization of the sacral vertebral bodies and anterior longitudinal ligament. The OPAL2 dataset includes women with POP as well as age- and parity-matched controls without prolapse, allowing evaluation of presacral anatomy across a spectrum of pelvic floor support rather than as a formal case–control comparison. Given the absence of a standardized approach for presacral space measurement on MRI, we collaborated with a radiologist (JS) specializing in pelvic floor imaging to establish an optimal measurement method.

We determined that the anterior margin of the presacral space would be defined by the first intensity change rather than the posterior aspect of the rectum, as employed in previous studies [8]. Measurements conducted in this study encompassed the anterior disc space height between L5 and S1, vertebral height of S1–S3, presacral depth and ALL thickness at vertebral bodies S1–S3 and the sacral promontory (SP).

**Fig. 1:**
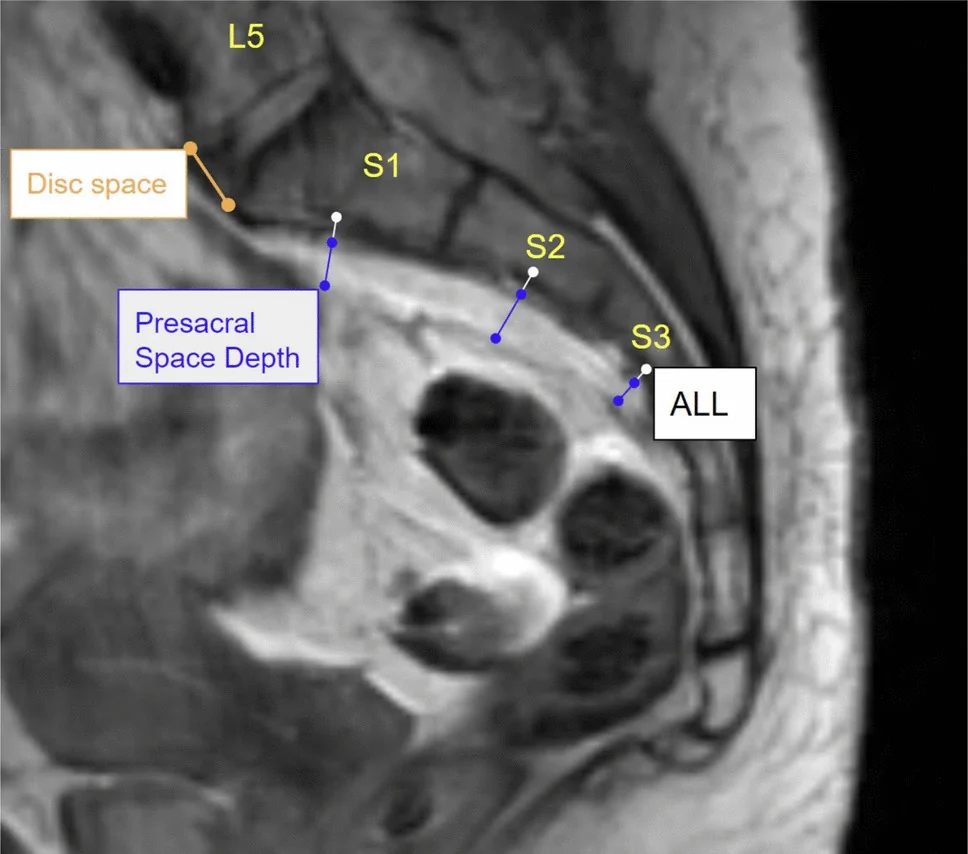
Mid-sagittal pelvic MRI with example measurements for presacral space depth, ALL and L5-S1 disc space measurements. ALL Anterior longitudinal ligament. (See attached picture)

Presacral depth at each vertebral level was calculated by identifying the anterior midpoint of each vertebral body and measurement was made to the first radiographic intensity change observed anterior to the ALL (Fig. 1). Because the peritoneum itself is not consistently visualized on standard pelvic MRI sequences, the first radiographic intensity change anterior to the ALL was used as a reproducible imaging landmark approximating the posterior peritoneal boundary. This landmark was consistently identifiable across scans and provided a standardized reference point for measurement. Simultaneously, ALL thickness was measured along this same plane. To ensure robustness and reliability, measurements were performed by three independent raters from the Society of Gynecologic Surgeons Pelvic Anatomy Group. Each image was independently evaluated by two of the three raters, and measurement reproducibility was assessed using inter-rater reliability coefficients. Raters were blinded to each other’s measurements and to patient demographics.

Data processing and statistical analysis were performed in Python. Descriptive statistics were calculated for patient characteristics and anatomic measurements. Pearson correlation coefficients were used to evaluate associations between continuous variables and presacral depth, while group comparisons were performed using two-sample *t*-tests. Effect sizes were calculated using Cohen’s *d*. Inter-rater reliability was calculated using an intraclass correlation coefficient.

## Results

A total of 238 pelvic MRIs were reviewed. Of these, 209 were included for measurement after excluding 29 studies due to incomplete visualization of relevant anatomy. Patient demographic data were available for 167 MRIs and were included in statistical analysis (Table 1). Sacral and presacral measurements are summarized in Table 2.

**Table 1:** Patient demographics

Demographics (*n* = 167)	
	Mean ± SD
Age (years)	58.3 ± 10.8
Parity (births)	2.7 ± 1.4
BMI (kg/m^2^)	26.9 ± 5.2 (range 18.5–44.7)
Stage of BMI	Percentage of patients
Underweight < 18.5	0 (0%)
Normal weight BMI 18.5 to 24.9	72 (43%)
Overweight – BMI greater than or equal to 25 to 29.9 kg/m^2^	57 (34%)
Obesity class I – BMI 30 to 34.9 kg/m^2^	25 (15%)
Obesity class II – BMI 35 to 39.9 kg/m^2^	12 (7%)
Obesity class III – BMI greater than or equal to 40 kg/m^2^	1 (1%)
	Responded yes (%)
Menopausal status	112 (67%)
Exogenous estrogen use	20 (12%)
Prior hysterectomy	33.4 (20%)
Smoker	7 (4%)
Constipation	32 (19%)
Race	145 (87%) White 3 (2%) Hispanic 12 (7%) African- American 3 (2%) Asian 4 (2%) Other
POP Q stage of prolapse	Percentage of patients
0	5 (3%)
1	22 (13%)
2	67 (40%)
3	68 (41%)
4	5 (3%)

**Table 2:** Sacral measurements

Sacral measurements (*n* = 209)		
		Mean ± SD mm
L5–S1 disc space		13.8 ± 3.7
SP	ALL	2.6 ± 0.8
	PSS	3.7 ± 1.5
S1	ALL	2.5 ± 0.8
	PSS	4.5 ± 2.2
	Vertebral height	31.2 ± 2.3
S2	ALL	2.4 ± 0.8
	PSS	4.8 ± 1.7
	Vertebral height	25.3 ± 2.5
S3	ALL	2.4 ± 0.7
	PSS	4.7 ± 1.7
	Vertebral height	20.0 ± 2.5

*SP* sacral promontory, *ALL* anterior longitudinal ligament, *PSS* presacral space depth

Correlation and subgroup analyses were performed to evaluate associations between patient characteristics and presacral anatomic measurements. Correlation analyses demonstrated weak correlations between BMI and presacral space depth across sacral levels, with Pearson correlation coefficients ranging from *r* = 0.20 to 0.25. Similarly, presacral depth did not differ significantly across other evaluated patient characteristics, including age, race, parity, smoking status, constipation, menopausal status, or POP diagnosis (all *p* > 0.05). In contrast, patients with a history of hysterectomy demonstrated a 24% greater presacral depth at S1 compared with those with an intact uterus (*p* = 0.01, Cohen’s *d* = 0.5). Postmenopausal status was associated with a 9% increase in anterior L5–S1 disc space height (*p* = 0.03, *d* = 0.4), and exogenous estrogen use was associated with a 13% increase in anterior L5–S1 disc height (*p* = 0.02, *d* = 0.4). Presacral depth was positively correlated with S1 vertebral body height (*r* = 0.40) and S2 vertebral body height (*r* = 0.48), both demonstrating moderate correlations.

Presacral space depth was significantly shallower at the sacral promontory compared with S1, S2 and S3 (Table 2). Conversely, ALL thickness was greatest at the sacral promontory. No meaningful differences in presacral space depth or ALL thickness were observed between the remaining sacral levels. Inter-rater reliability for sacral measurements demonstrated moderate agreement, with a mean intraclass correlation coefficient (ICC) of 0.67 (range 0.46–0.94).

## Discussion

This MRI-based study demonstrated that presacral depth in women is not meaningfully associated with BMI, while a history of hysterectomy was associated with increased presacral depth at S1. Additionally, presacral depth was consistently shallower at the sacral promontory compared to more caudal sacral levels, and ALL thickness was greatest at the promontory. Presacral depth was also moderately correlated with sacral vertebral body height, suggesting that underlying vertebral morphology may contribute to variation in presacral anatomy. Together, these findings provide clinically relevant anatomic insight for surgeons performing procedures requiring presacral dissection.

The absence of an association between BMI and presacral depth aligns with prior literature demonstrating that BMI does not reliably reflect the distribution of adipose depots, particularly visceral or perivascular adipose tissue in older adults [9]. Because presacral depth reflects a retroperitoneal anatomic relationship influenced by local connective tissue and perivascular fat rather than subcutaneous adiposity, BMI would not be expected to reliably predict variation in this space. These findings suggest that BMI alone should not be used to anticipate technical complexity of the presacral dissection.

The observed increase in presacral depth among patients with prior hysterectomy may reflect postsurgical anatomic remodeling. While the mechanism underlying this association remains unclear, it raises the possibility that prior pelvic surgery may influence presacral anatomy. Further investigation is needed to determine whether similar changes occur following pelvic procedures.

Our sacral measurements are generally consistent with prior cadaveric and imaging-based studies. Florian et al. reported a mean anterior longitudinal ligament thickness at S1 of 1.9 mm in cadaveric specimens, compared with 2.5 mm in this in vivo MRI study [10]. Differences between cadaveric and imaging measurements may reflect several factors, including tissue preservation effects, physiologic hydration of living tissues, and age-related degenerative changes of the spine that can alter disc and ligament morphology. From a surgical standpoint, this finding is reassuring since a thicker ALL provides a more robust site for mesh fixation. This is further supported by evidence demonstrating that apical failure after sacrocolpopexy more commonly results from vaginal detachment of the mesh rather than failure at the sacral fixation point [11].

The anterior L5–S1 disc height measured in this study was slightly smaller than values reported in prior cadaveric investigations. This difference may reflect methodological differences between studies. In this analysis, disc height was measured at the anterior margin of the disc using midsagittal MRI, whereas many cadaveric studies report overall disc height, including both anterior and posterior dimensions. Additionally, in vivo imaging reflects physiologic loading and tissue hydration that may differ from cadaveric specimens. For these reasons, direct comparison of absolute disc height values between imaging and cadaveric studies should be interpreted cautiously [10]. This measurement approach was selected because the anterior disc space is most clinically relevant during sacrocolpopexy when placing sutures into the ALL while avoiding the disc space [12].

Postmenopausal state and exogenous estrogen use were associated with increased anterior L5–S1 disc space height in our analysis. This finding differs from prior studies reporting decreased intervertebral disc height with aging and menopause, which is generally attributed to degenerative disc changes. However, most prior investigations evaluate overall disc height using combined anterior and posterior measurements, whereas our measurements focused exclusively on the anterior disc space because of its surgical relevance during sacrocolpopexy [13, 14]. Additionally, the observed association may reflect differences in patient selection, imaging technique, or unmeasured confounders within this retrospective dataset. As disc height was not the primary focus of this study, this finding should be interpreted cautiously and warrants further investigation.

Given that estrogen receptors are present in lumbar intervertebral discs and estrogen has been shown to promote annulus cell proliferation, these findings warrant further investigation but were not the primary focus of this study [13].

Presacral space depth was shallowest at the sacral promontory, while ALL thickness was greatest at this level. Although this may facilitate safer retroperitoneal entry, the sacral promontory is not typically considered the ideal fixation site due to the risk of discitis and unfavorable vaginal axis. Prior studies have demonstrated that fixation at more caudal levels better restores the native vaginal access (found to angle to S3–S4), although hemorrhage risk increases with deeper dissection [15–17]. Fixation closer to the promontory has been advocated to improve visualization of the middle sacral vessels [11]. A cadaveric study demonstrated that fixation at the lower border of S2 compared with the lower border of L5 resulted in a threefold increase in vaginal axis angle [17]. While the ideal fixation site remains debated, there is consensus that the disc space should be avoided to reduce the risk of discitis [12]. On the bases of our findings, safe fixation can be achieved either inferior to the sacral promontory or superior to the promontory above the disc space, which had a mean height of 1.4 cm (range 0.5–2.8 cm), with larger measurements reported in cadaveric studies [12]. If S2 is the intended fixation level, our data suggest that dissection greater than 3 cm caudal to the promontory is required to extend beyond the mean S1 vertebral height.

Despite evidence that obesity increases overall perioperative risk, this study does not support the use of BMI as a predictor of presacral dissection complexity [18, 19]. Given the well-documented presence of anti-obesity bias in surgical practice, these findings underscore the importance of avoiding assumptions about anatomic difficulty based on BMI alone [20].

Strengths of this study include its large MRI cohort, use of blinded independent raters, and integration of imaging with clinically relevant patient characteristics. Although the cohort represents one of the larger MRI-based analyses of presacral anatomy, limitations include selection bias inherent to the source database, limited racial diversity, and the absence of standardized radiologic definitions of presacral depth. Additionally, prior pelvic surgeries other than hysterectomy and inflammatory conditions were not captured and may represent areas for future study.

In conclusion, presacral space depth was not associated with BMI but was modestly increased in patients with prior hysterectomy, while other evaluated patient characteristics, including age, race, parity, and prolapse status, were not significantly associated with presacral depth. These findings suggest that variation in presacral anatomy is more closely related to underlying anatomic and surgical factors than body habitus alone. As preoperative pelvic imaging becomes increasingly integrated into surgical planning for prolapse repair, assessment of presacral anatomy may provide valuable insight into operative risk and optimal sacral fixation strategies.

## Data Availability

The data that support the findings of this study are available from the corresponding author upon reasonable request.
